# Supplementary material for: Translating area-based conservation pledges into efficient biodiversity protection outcomes
Source: Commun Biol. 2021 Sep 7;4:1043. doi: 10.1038/s42003-021-02590-4 (PMC8423728; doi:10.1038/s42003-021-02590-4)
Supplement: Supplementary file 2 — Supplementary information [file 42003_2021_2590_MOESM2_ESM.pdf]

Translating area-based conservation pledges into efficient biodiversity protection outcomes

Charles A. Cunningham[1]*, Humphrey Q. P. Crick[2], Mike D. Morecroft[3], Chris D. Thomas[1*†], Colin M. Beale[1,4*†]

[1]Leverhulme Centre for Anthropocene Biodiversity, Department of Biology, University of York, York, YO10 5DD, UK

[2] Natural England, Eastbrook, Shaftesbury Road, Cambridge, CB2 8DR

[3] Chief Scientist's Directorate, Natural England % Natural England Mail Hub, Worcester, WR5 2NP.

[4] York Environmental Sustainability Institute, University of York, York, YO10 5DD, UK

*Corresponding author

†These authors contributed equally

Correspondence:

CAC: cac567@york.ac.uk

HQPC: humphrey.crick@naturalengland.org.uk

MDM: mike.morecroft@naturalengland.org.uk

CDT: chris.thomas@york.ac.uk

CMB: colin.beale@york.ac.uk

Supplementary Materials

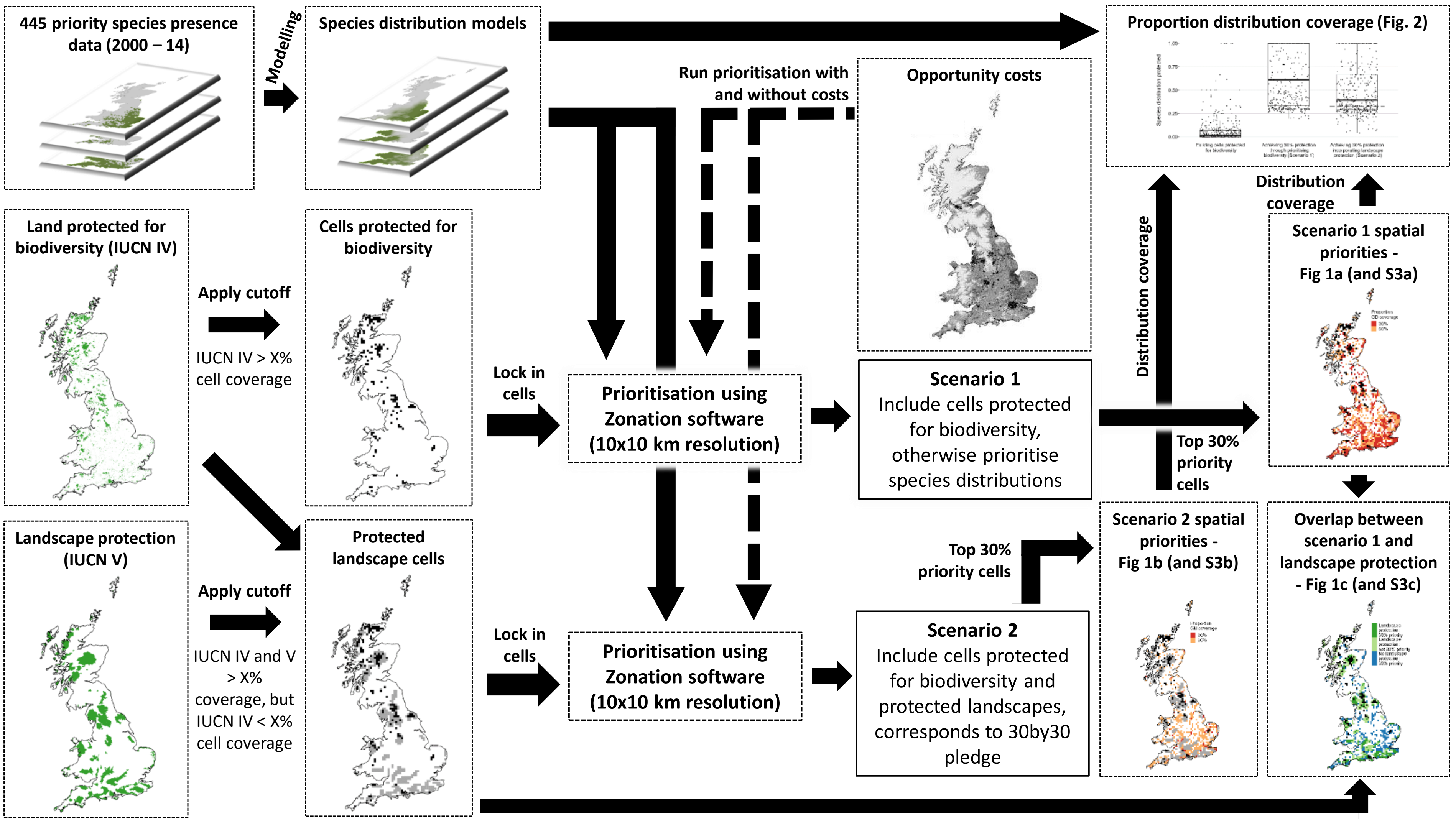

Supplementary Figure 1 Approach workflow for evaluating different conservation scenarios in terms of coverage of species distributions. We firstly categorised conservation designations into two types; 'protection for biodiversity' (IUCN IV or higher protection), and 'landscape protection' (lower level IUCN V protection). SSSIs and NNRs are 'protected for biodiversity', and NPs, AONBs, and Scottish NSAs offer landscape protection. We then used these to classify 10x10km cells using several cutoffs (Supplementary Table 1). If cells had greater 'land protected for biodiversity' coverage than the cutoff, they were classified as 'protected for biodiversity' cells. Otherwise if cells met the cutoff with a combination of land 'protected for biodiversity' and landscape protection, but were not 'protected for biodiversity' cells, then they were classified as 'protected landscape' cells. We also used the recorded distributions of 445 priority species between 2000 and 2014. These distributions were modelled using INLA (See Supplementary Methods) to interpolate distributions of less recorded species. The species distributions and protected cells were then used for two spatial prioritisations exploring different conservation scenarios. Scenario 1 required inclusion of 'protected for biodiversity' cells but didn't consider other designations beyond that. Scenario 2 also included cells 'protected for biodiversity' but, corresponding to the 30by30 pledge, additionally required all protected landscapes to be included in the solution. All prioritisations were undertaken at a 10x10 km landscape-scale on cells with greater than half land coverage. The spatial prioritisations were carried out using Core Area Zonation, whereby cells are removed iteratively, and cells remaining longer within the solution complement species representation of other cells to a greater extent. Cells with the lowest value are removed first, corresponding to the lowest maximum proportion of species distributions within the remaining cells. Priorities were constrained by masking or 'locking in' different areas relevant to each scenario such that all other cells must be removed first. In order to compare the scenarios, we calculated the proportion of each species distribution covered by the top 30% priority cells of each scenario (Fig. 2, Supplementary Table 2). Finally, we compared the spatial overlap of protected landscape cells and scenario 1 30% priorities (Fig 1c, Supplementary Table 3). We also undertook a parallel analysis additionally incorporating opportunity costs calculated from agricultural land classification and urban

areas (Supplementary Fig. 2, Supplementary Table 4). In this analysis, cell value was divided by the mean opportunity cost of the cell (Supplementary Fig. 3, Supplementary Tables 2 and 3).

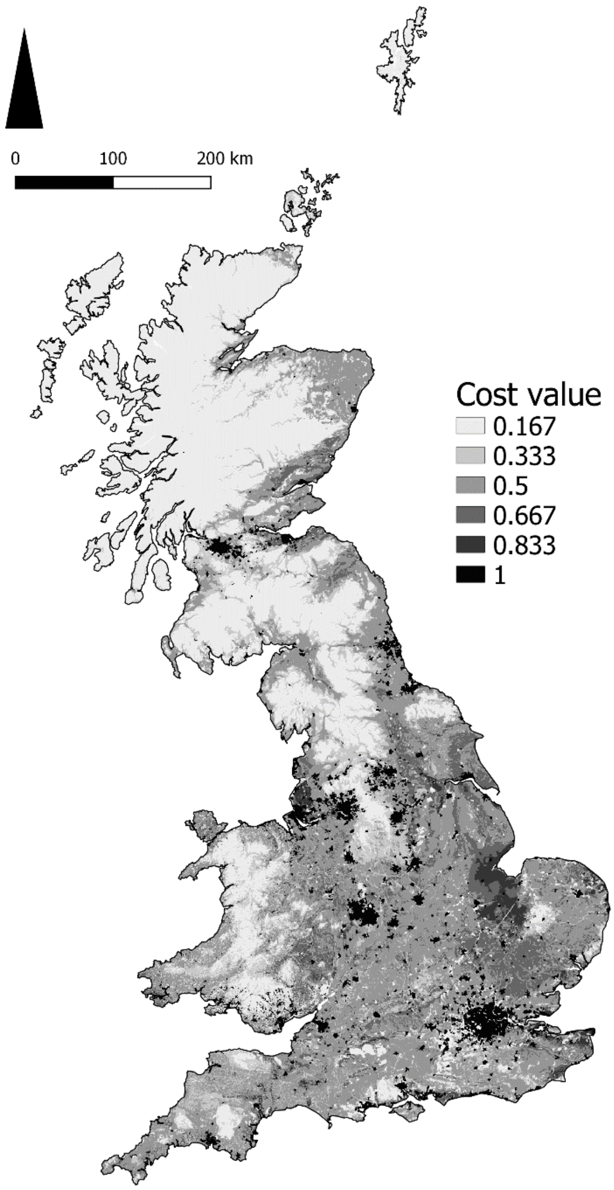

Supplementary Figure 2 Agricultural land classifications used as a proxy for opportunity cost in spatial prioritisations. Opportunity costs were assigned based upon agricultural land classifications for England, Scotland, and Wales. Agricultural land classification was standardised between countries, then rescaled and subtracted from 1 as presented in Supplementary Table 4. Urban land was then given the largest possible cost value of 1. Costs were aggregated by mean cell cost for prioritisations undertaken at 10x10 km resolution.

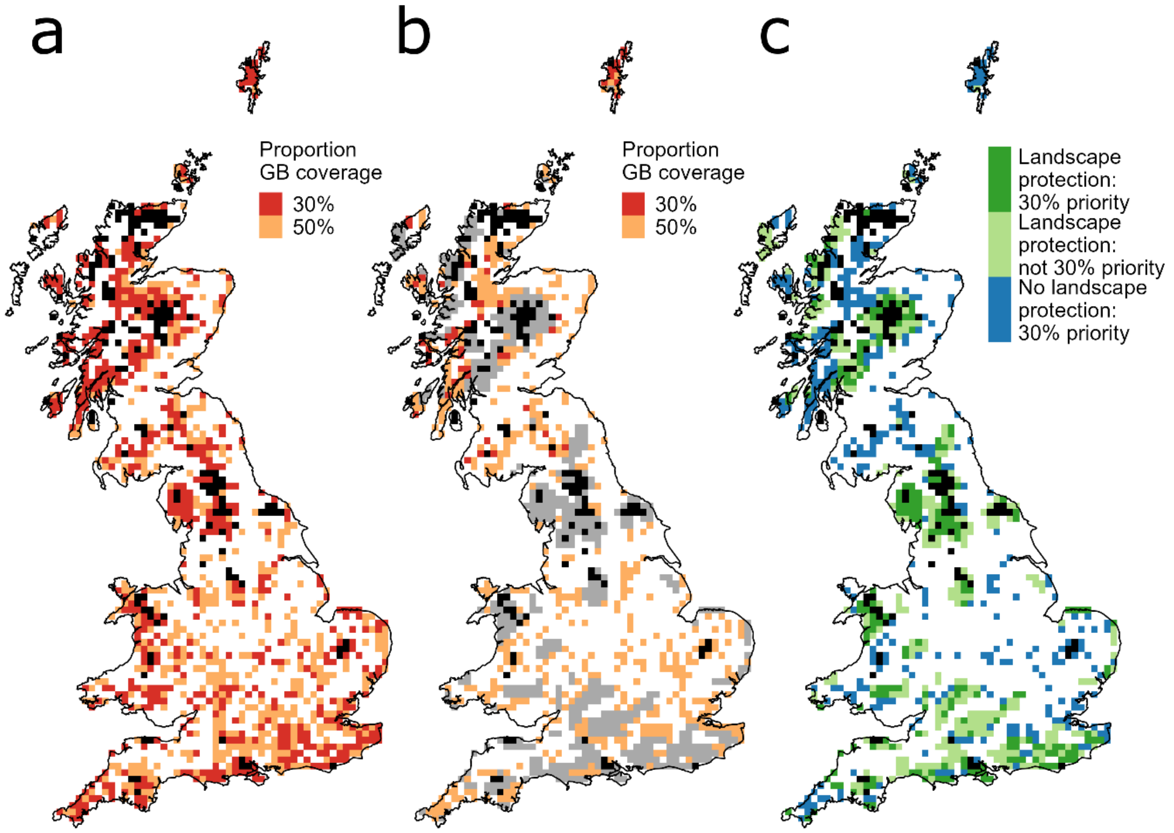

Supplementary Figure 3 Spatial priorities for additional protection to meet 30by30 protection targets within Britain incorporating opportunity costs into the spatial prioritisation. (a) Scenario 1: prioritisation constrained only by the inclusion of current biodiversity protected sites. (b) Scenario 2: constrained by maintaining both biodiversity and landscape protection sites, as suggested by the 30by30 announcement. (c) Overlap between top 30% priority cells for biodiversity from scenario 1 and current protected landscapes. Cells already protected for biodiversity are shaded black (which are included as part of the ‘top 30% in both scenarios). For panels (a) and (b), top 30% priority cells are shaded red, top 50% orange, and landscape protection cells are grey. In panel (c), priority cells for biodiversity are dark green if in a landscape protection cell and dark blue if outside a landscape protection cell; light green shows those landscape protected cells that are not a priority for biodiversity conservation.

Supplementary Table 1 Number of cells within each protection category for different protection level cutoffs, with percentages of total 2309 GB cells in parentheses. We considered land protected for biodiversity to include Sites of Special Scientific Interest (SSSI) and National Nature Reserves (NNR) (9.04% actual proportion GB coverage); and landscape protection to include National Parks (NP), Areas of Outstanding Natural Beauty (AONB), and Scottish National Scenic Areas (NSA) (22.22% actual proportion GB coverage) [intersection between categories 4.55%, union 26.71%]. 10x10 km cells listed below were considered to be 'protected for biodiversity' if SSSI/NNR coverage was greater than the cutoff proportion of the land area, i.e. at least X% IUCN minimum IV protection. 'Protected landscapes' contained total coverage from all of the designations > X%, i.e. at least X% minimum IUCN V protection, but the cell was not already 'protected for biodiversity'. *At the 30% protection cutoff more than 30% of GB cells are protected for biodiversity and protected landscapes at 10x10 km resolution.

	Minimum proportion of each cell currently protected				
	**30%***	**40%**	**50%**	**60%**	**70%**
**Protected for biodiversity**	211 (9.14%)	148 (6.41%)	99 (4.29%)	61 (2.64%)	27 (1.17%)
**Protected landscapes**	531 (23.00%)	494 (21.39%)	459 (19.88%)	404 (17.50%)	362 (15.68%)
**Total**	742 (32.14%)	642 (27.80%)	558 (24.17%)	465 (20.14%)	389 (16.85%)

Supplementary Table 2 Median proportion of priority species distributions protected under different scenarios. For each current protection cutoff, and with/without inclusion of opportunity costs, the median species protected is presented for: *Scenario 1,* existing land protected for biodiversity and including additional cells on the basis of the distributions of priority species, unconstrained by current landscape protection, and; *Scenario 2*, includes existing cells protected for biodiversity *and* those with current landscape protection, before including additional cells on the basis of the distributions of priority species. For scenario 2, the median proportion covered by cells already protected for biodiversity and protected landscapes is included in parentheses. Scenario 1 consistently had median protection at least 10% higher than scenario 2 protecting 30% of land. *At the 30% protection cutoff more than 30% of GB cells are protected for biodiversity and protected landscapes at 10x10 km resolution.

		**Median proportion priority species distribution protected (%)**			
		**Not including costs**		**Including costs**	
**Minimum proportion of each cell currently protected**	**Cells**	**30% GB coverage**	**50% GB coverage**	**30% GB coverage**	**50% GB coverage**
**30%***	Current land protected for biodiversity	3.96			
	Scenario 1<br>(includes cells already protected for biodiversity)	58.74	94.44	45.45	79.18
	Scenario 2<br>(includes cells already protected for biodiversity and protected landscapes)	-<br>(34.75)	83.33<br>(34.75)	-<br>(34.75)	76.02<br>(34.75)
**40%**	Current land protected for biodiversity	1.63			
	Scenario 1	61.18	96.23	48.39	81.28
	Scenario 2	39.32<br>(29.47)	90.31<br>(29.47)	31.30<br>(29.47)	76.47<br>(29.47)
**50%**	Current land protected for biodiversity	0.66			
	Scenario 1	65.04	97.71	50.00	81.28
	Scenario 2	48.52<br>(24.87)	92.23<br>(24.87)	33.33<br>(24.87)	75.33<br>(24.87)
**60%**	Current land protected for biodiversity	0.41			
	Scenario 1	68.19	98.12	50.00	81.77
	Scenario 2	55.14<br>(19.72)	93.79<br>(19.72)	37.44<br>(19.72)	77.40<br>(19.72)
**70%**	Current land protected for biodiversity	0.00			
	Scenario 1	66.13	98.48	50.00	82.26
	Scenario 2	55.56<br>(15.38)	93.87<br>(15.38)	40.00<br>(15.38)	79.18<br>(15.38)

Supplementary Table 3 Cell overlap between top 30% priority cells from scenario 1 (prioritising cells to add to existing cells protected for biodiversity unconstrained by current landscape protection), and protected landscape cells. Number of cells in three categories are presented; cells with landscape protection that are a 30% priority, cells with landscape protection that are not a 30% priority, and cells without landscape protection (or protection for biodiversity) that are a 30% priority. Of cells not protected for biodiversity, the majority of scenario 1 30% priority cells were outside protected landscapes, and a large proportion of protected landscape cells were not a 30% priority. Additionally, the mean proportion of land currently protected for biodiversity within each category is presented. 30% priority cells outside protected landscapes consistently had the lowest amount of land protected for biodiversity. Results presented for all protection cutoffs tested, both with costs (Fig. 1c) and without (Supplementary Fig. 3c).

Minimum proportion of each cell currently protected	Cells	Not including costs		Including costs	
		No. of cells	% protected	No. of cells	% protected
30%	Landscape protection: 30% priority	196	7.77	197	9.12
	Landscape protection: 30% non-priority	335	8.17	334	7.38
	No landscape protection: 30% priority	286	4.33	285	4.58
40%	Landscape protection: 30% priority	205	10.27	204	12.10
	Landscape protection: 30% non-priority	289	10.36	290	9.07
	No landscape protection: 30% priority	340	4.77	341	5.55
50%	Landscape protection: 30% priority	203	12.68	207	15.00
	Landscape protection: 30% non-priority	256	13.38	252	11.49
	No landscape protection: 30% priority	391	5.10	387	6.50
60%	Landscape protection: 30% priority	187	14.8	197	17.67
	Landscape protection: 30% non-priority	217	16.81	207	14.18
	No landscape protection: 30% priority	445	5.70	435	7.63
70%	Landscape protection: 30% priority	175	17.71	187	19.79
	Landscape protection: 30% non-priority	187	19.04	175	16.91
	No landscape protection: 30% priority	491	6.83	479	8.55

Supplementary Table 4 Agricultural land classifications within England, Scotland, and Wales. Classifications were standardised between nations into a single interoperable agricultural land value code. We rescaled these values and subtracted from 1 to calculate opportunity cost. Urban land was given the largest possible opportunity cost value (1).

England Code	Wales Code	England/Wales Description	England/ Wales Detail	Scotland Code	Scotland Description	Interoperable Code	Opportunity cost used in prioritisation
**Grade 1**	1	Excellent quality	No or very minor limitations on agricultural use. Wide range of agricultural and horticultural crops can be grown. High yielding and consistent.	1	Land capable of producing a very wide range of crops	1	0.833
**Grade 2**	2	Very good	Minor Limitations on crop yield, cultivations or harvesting. Wide range of crops but limitations on demanding crops (e.g. winter harvested veg). Yield high but lower than Grade 1.	2	Land capable of producing a wide range of crops	2	0.666
**Grade 3**	3a	Good	Moderate to high yields of narrow range of arable crops (e.g. cereals), or moderate yields of grass, oilseed rape, potatoes, sugar beet and less demanding horticultural crops	3.1	Land capable of producing consistently high yields of a narrow range of crops and/ or moderate yields of a wider range. Short grass leys are common	3	0.500
**Grade 3**	3b	Moderate	Moderate yields of cereals, grass and lower yields other crops. High yields of grass for grazing/ harvesting.	3.2	Land capable of average production though high yields of barley, oats and grass can be obtained. Grass leys are common		
**Grade 4**	4	Poor	Severe limitations which restrict range and/or level of yields. Mostly grass and occasional arable (cereals and forage), but highly variable yields. Very droughty arable land included.	4.1	Land capable of producing a narrow range of crops, primarily grassland with short arable breaks of forage crops and cereal	4	0.333
				4.2	Land capable of producing a narrow range of crops, primarily on grassland with short arable breaks of forage crops		

**Grade 5**	5	Very poor	Severe limitations which restrict use to permanent pasture or rough grazing except for pioneering forage crops.	5.1	Land capable of use as improved grassland. Few problems with pasture establishment and maintenance and potential high yields	5	0.167
				5.2	Land capable of use as improved grassland. Few problems with pasture establishment but may be difficult to maintain		
				5.3	Land capable of use as improved grassland. Pasture deteriorates quickly		
				6.1	Land capable of use as rough grazings with a high proportion of palatable plants		
				6.2	Land capable of use as rough grazings with moderate quality plants		
				6.3	Land capable of use as rough grazings with low quality plants		
				7	Land of very limited agricultural value		
**Non Agricultural**	NA	Non-agricultural		999	Inland Water		
**Exclusion**	NA	Non-agricultural		9500	Unencoded Islands		
**Urban**	U	Urban		888	Built Up Areas	0	1.000

Supplementary Methods - Species Distribution Models

For our models, we used the recorded distributions of 445 priority species listed under Section 41 (Natural Environment and Rural Communities Act, 2006), provided by Butterfly Conservation (BC), Biological Records Centre (BRC); and breeding bird atlas data from British Trust for Ornithology (BTO)[24]. Species distributions were in the form of annual presence records that were aggregated together for modelling between 2000 and 2014, apart from birds and vascular plants which were only available for specific time periods (2007-11, and 2010-17 respectively). Input data used was in the form of presence/ pseudo-absence at 10x10km scale, and we only used species with spatial records present in <50% land coverage cells.

Of the 445 total priority species, 156 species were very localised (10 or fewer presence records), and deemed unsuitable for modelling over the whole of Britain, hence we used the raw distribution records for prioritisations.

For the other species which had over 10 presence records, we carried out modelling individually for each species to interpolate their range using Integrated Nested Laplace Approximations (INLA) in the inlabru R package[25]. A joint model predicting distribution while accounting for recording effort was used (Eq. 1), including biologically relevant covariates: seasonality (cvTemp), the coefficient of temperature variation; growing degree days (GDD5), the number of days 5°C or warmer as a measure of the plant growth season; water availability (water), calculated using rainfall and evapotranspiration as well as soil moisture; and winter cold (MTCO), the mean temperature of the coldest month. See Beale et al. (2014) for calculation methods of these covariates. These covariates were calculated using monthly means of weather data for 2004-2014, specifically mean temperature, sunshine and rainfall from the Met Office[28]. We also included soil moisture to calculate water availability[29]. Additionally we included soil PH, which was aggregated from 1x1 km resolution soil pH from the Countryside Survey 2007 dataset to 10x10km cells using the mean.

To estimate recorder effort, we used the raw species distribution data records from all 445 species. These were used, along with broad habitat layers extracted from the Land Cover Map 2015[30], in a Frescalo analysis. Frescalo works through a number of stages to estimate recorder effort, but see Hill (2012) for further details on use of Frescalo software. Simply, for each cell a matrix of weights is created for neighbouring cells, with higher weights for spatial proximity and habitat similarity. Species presences are then multiplied by these weights, and recorder effort is estimated based upon the difference between the focal cell value and the neighbourhood mean cell value. A hazard rate detection function was then used (Eq. 2) to estimate the effect of recorder effort on recorded species presence for each species. The models of 77 species for which modelling was attempted did not converge (most of which were very rare), and so the raw distributions were used in these instances.

$$\log(\lambda_i) = b_0 + b_1 cvTemp_i + b_2 cvTemp_i{}^2 + b_3 GDD5_i + b_4 GDD5_i{}^2 + b_5 water_i + b_6 water_i{}^2 + b_7 MTCO_i + b_8 MTCO_i{}^2 + b_9 PH_i + b_{10} PH_i{}^2 + \log(p_i) + SE_i$$

Eq. 1

where λ_i is the spatial intensity (density) of species presence at cell *i*, b_0 is the intercept, *cvTemp* is seasonality, *GDD5* is growing degree days, *water* is water availability, *MTCO* is winter cold, *PH* is the soil PH, p_i is the detection probability function, SE_i is the structured and random spatial effect for cell *i*, and $b_{1\text{-}10}$ are the estimated parameters for the corresponding covariates.

$$p_i = 1 - e^{-\left(\frac{d_i}{e^{\hat{\sigma}}}\right)^{-1}}$$

Eq. 2

where d_i is the inverse of estimated recorder effort at cell *i,* and $\hat{\sigma}$ is the linear predictor of the sigma parameter.
