# Supplementary material for: Translating area-based conservation pledges into efficient biodiversity protection outcomes
Source: Commun Biol. 2021 Sep 7;4:1043. doi: 10.1038/s42003-021-02590-4 (PMC8423728; doi:10.1038/s42003-021-02590-4)
Supplement: Supplementary file 3 — Description of Additional Supplementary Files [file 42003_2021_2590_MOESM3_ESM.pdf]

Description of Additional Supplementary Files

File name: Supplementary Data 1

Description: 428 species with spatial data available included within this study. Only species level complementarity was considered, and so where several sub-species are listed, these were then aggregated. 7 additional species could not be included in the prioritisations as they were only present in coastal cells with <50% land coverage (end of table).
